# Long-term efficacy and safety of early alogliptin initiation in subjects with type 2 diabetes: an extension of the SPEAD-A study

**DOI:** 10.1038/s41598-023-41036-1

**Published:** 2023-09-05

**Authors:** Tomoya Mita, Naoto Katakami, Hidenori Yoshii, Tomio Onuma, Hideaki Kaneto, Takeshi Osonoi, Toshihiko Shiraiwa, Tetsuyuki Yasuda, Yutaka Umayahara, Tsunehiko Yamamoto, Hiroki Yokoyama, Nobuichi Kuribayashi, Hideaki Jinnouchi, Masahiko Gosho, Iichiro Shimomura, Hirotaka Watada

**Affiliations:** 1 Department of Metabolism and Endocrinology, Juntendo University Graduate School of Medicine, Hongo 2-1-1, Bunkyo-Ku, Tokyo, 113-8421 Japan; 2 Department of Metabolic Medicine, Osaka University Graduate School of Medicine, 2-2, Yamadaoka, Suita, Osaka 565-0871 Japan; 3 Department of Medicine, Diabetology and Endocrinology, Juntendo Tokyo Koto Geriatric Medical Center, Shinsuna 3-3-20, Koto-Ku, Tokyo, 136-0075 Japan; 4 Department of Diabetes, Endocrinology and Metabolism, Kawasaki Medical School, 577 Matsushima, Kurashiki, Okayama 701-0192 Japan; 5 Naka Kinen Clinic, 745-5, Nakadai, Naka City, Ibaraki 311-0113 Japan; 6 Shiraiwa Medical Clinic, 1-12-8 Hirano, Kashiwara, Osaka 582-0019 Japan; 7 Osaka Police Hospital, 10-31 Kitayamacho, Tennoji-Ku, Osaka 543-0035 Japan; 8 Osaka General Medical Center, 3-1-56 Bandai-Higashi, Sumiyoshi-Ku, Osaka 558-8558 Japan; 9 Kansai Rosai Hospital, 3-1-69 Inabasou, Amagasaki-Shi, Hyogo 660-8511 Japan; 10 Jiyugaoka Medical Clinic, Internal Medicine, West 6, South 6-4-3, Obihiro, Hokkaido 080-0016 Japan; 11 Misaki Naika Clinic, 6-44-9 Futawahigashi, Funabashi, Chiba 274-0805 Japan; 12 Jinnouchi Hospital, Kuhonji 6-Chome 2-3, Kumamoto, 862-0976 Japan; 13 Department of Biostatistics, Institute of Medicine, University of Tsukuba, 1-1-1, Tennodai, Tsukuba, Ibaraki 305-8575 Japan

**Keywords:** Endocrinology, Medical research

## Abstract

We previously reported in the study of preventive effects of alogliptin on diabetic atherosclerosis (SPEAD-A) that alogliptin, a dipeptidyl peptidase-4 (DPP-4) inhibitor, attenuated the progression of carotid atherosclerosis in subjects with type 2 diabetes and no history of cardiovascular disease. This extension study of the SPEAD-A trial investigated whether early alogliptin initiation improved long-term cardiovascular outcomes. The SPEAD-A trial randomized 341 subjects with type 2 diabetes to either alogliptin or conventional treatment to investigate the effects of alogliptin on atherosclerosis. All subjects who completed that trial were eligible for this prospective, observational cohort study. The primary endpoint was the first occurrence of a major cardiovascular event, defined as death due to any cause, acute myocardial infarction, or stroke. During the 520-week follow-up period, composite primary outcome events occurred in only a few subjects in each group [8 (5.4%) in the alogliptin group and 9 in the conventional treatment group (5.9%)]. There were no significant differences in the incidence rate of the primary outcome between the two groups. Post hoc Poisson regression analysis showed no significant difference between the two groups in the incidence rate of composite recurrence events for the same outcomes as the primary endpoint. On the other hand, this incidence rate was significantly lower in subjects who received DPP-4 inhibitors before an initial cardiovascular event than in those who did not (5.8 vs. 13.3 per 1000 person-years, respectively, *p* = 0.04). Early initiation of alogliptin was not associated with a reduced risk of composite cardiovascular disease, which could be attributed to fewer events and/or the addition of DPP-4 inhibitors during the follow-up period.

## Introduction

People with type 2 diabetes mellitus are at high risk of cardiovascular disease (CVD), which undermines quality of life and reduces life expectancy^1^. Thus, one of the main goals of type 2 diabetes management is to prevent or at least delay cardiovascular events by achieving and sustaining glycemic targets and managing cardiovascular risk factors.

In recent years, different classes of glucose-lowering agents have been developed, including dipeptidyl peptidase-4 (DPP-4) inhibitors, sodium-glucose cotransporter 2 (SGLT-2) inhibitors, and glucagon-like peptide-1 (GLP-1) receptor agonists, thus expanding the range of treatments options for type 2 diabetes. The United States Food and Drug Administration requires experimental evidence that new classes of glucose-lowering agents do not cause an unacceptable increase in cardiovascular risk. Five recent cardiovascular outcome trials (CVOTs) showed that DPP-4 inhibitors were not inferior to placebo^2–5^ or to sulfonylureas^6^, suggesting their cardiovascular safety. However, these trials did not demonstrate superior prevention of cardiovascular events in people with type 2 diabetes and a history of CVD or a high risk of CVD. In contrast, emerging evidence from several CVOTs indicates that SGLT-2 inhibitors^7–9^ and GLP-1 receptor agonists^10,11^ may reduce the risk of the composite of death, myocardial infarction, and stroke in people with type 2 diabetes and a history of CVD or a high risk of CVD. Based on these data, current guidelines developed by the American Diabetes Association recommend the use of SGLT-2 inhibitors and/or GLP-1 receptor agonists as components of glucose-lowering regimens and to achieve comprehensive cardiovascular risk reduction, with consideration of factors specific to each individual^12^.

Similarly, a recently published consensus statement by the Japan Diabetes Society recommends choosing therapies based on multiple factors, including aspects of each individual’s pathophysiological condition, for instance insufficient insulin secretion or insulin resistance^13^. In particular, East Asian people with type 2 diabetes usually exhibit lower insulin secretory capacity and lesser obesity than Caucasians. In addition, DPP-4 inhibitors provide more effective glycemic control in East Asian people than in Western people (16,17). Reflecting these pathophysiological differences, DPP-4 inhibitors are used extensively as first- or second-line therapies in Japan^14–17^. Among DPP-4 inhibitors, alogliptin was proven to have high selectivity for DPP-4^18^. In a real-world setting in Japanese people with type 2 diabetes, alogliptin demonstrated long-term efficacy and safety^19^. In addition, we previously conducted the Study of Preventive Effects of Alogliptin on Diabetic Atherosclerosis (SPEAD-A) trial, which showed that compared with conventional treatment, alogliptin treatment more effectively attenuated the progression of carotid intima-media thickness in subjects with type 2 diabetes and no apparent history of CVD. However, one of the limitations of the SPEAD-A trial was that it lacked sufficient power to detect differences in CVD outcomes between groups.

In this extension study of the SPEAD-A trial, we therefore examined whether the early initiation of DPP-4 inhibitors improved long-term clinical cardiovascular outcomes during a 520-week follow-up period.

## Results

### Participants’ demographic and background characteristics

Of the 302 subjects enrolled in this SPEAD-A Extension study, 149 and 153 were originally assigned to the alogliptin group and the conventional treatment group, respectively (Figure S1). There were no significant differences in their demographic or baseline characteristics (Table 1). Although a significantly higher number of subjects in the alogliptin group were receiving oral glucose-lowering agents at baseline, this difference disappeared by 26 weeks (Table S1). About 82% of subjects who had initially been assigned to receive alogliptin were receiving DPP-4 inhibitors at the end of study, while the percentage of those using alogliptin decreased from 100% at baseline to around 60% at end of the study. In contrast, approximately 63% of subjects who were originally allocated to the conventional treatment group were receiving DPP-4 inhibitors at the end of study. The percentage of subjects treated with oral glucose-lowering agents, including α-glucosidase inhibitors and glinides, was significantly higher in the conventional treatment group than in the alogliptin group at some points. Otherwise, there was no significant difference in the use of oral glucose-lowering agents between the two groups.

**Table 1:** Demographics and baseline characteristics of subjects of the two groups.

Parameters	Alogliptin treatment group (n = 149)	Conventional treatment group (n = 153)	*p* value
Gender (male) (%)	93 (62)	94 (61)	0.91
Age (years)	64.5 ± 9.9	65.1 ± 8.9	0.58
Current smoking	37 (25)	32 (21)	0.49
Hypertension	81 (54)	87 (57)	0.73
Systolic blood pressure (mmHg)	130 ± 16 (n = 149)	132 ± 15 (n = 153)	0.31
Diastolic blood pressure (mmHg)	75 ± 12 (n = 149)	75 ± 11 (n = 153)	0.83
Dyslipidemia	80 (54)	90 (59)	0.42
Total cholesterol at baseline (mmol/l)	4.99 ± 0.77 (n = 149)	5.00 ± 0.73 (n = 151)	0.91
HDL cholesterol at baseline (mmol/l)	1.46 ± 0.39 (n = 148)	1.42 ± 0.36 (n = 153)	0.30
Triglyceride at baseline (mmol/l)	1.17 (0.82,1.72) (n = 148)	1.24 (090,1.66) (n = 153)	0.40
Duration of diabetes (years)	9.0 (5.0 to 15.0)	10.2 (4.3 to 15.0)	0.76
Use of glucose-lowering agents			
Metformin	77 (52)	73 (48)	0.57
Sulfonylurea	74 (50)	88 (58)	0.20
Glinides	8 (5)	15 (10)	0.19
Thiazolidinediones	34 (23)	37 (24)	0.79
α-glucosidase inhibitors	51 (34)	47 (31)	0.54
Use of antihypertensive drugs			
Angiotensin-converting enzyme inhibitors	7 (5)	4 (3)	0.37
Angiotensin II receptor blockers	65 (44)	65 (42)	0.91
Calcium channel blockers	41 (28)	55 (36)	0.14
Use of lipid-lowering agents			
Statins	57 (38)	71 (46)	0.16
Fibrates	5 (3)	8 (5)	0.57
Use of anti-thrombotic agents			
Antiplatelet agents	18 (12)	21 (14)	0.73

Data are number (%) of patients, mean ± standard deviation, or median (quartiles 1 to 3).

### Changes in metabolic parameters during the study period

Relative to baseline, body mass index (BMI) decreased significantly after 260 weeks in the alogliptin group and after 104 weeks in the conventional treatment group (Table 2). The mean change in BMI was significantly larger in the alogliptin group than in the conventional treatment group at most observation points.

**Table 2:** Changes in body mass index and HbA1c level during the 520-week follow-up period.

Parameters	Alogliptin treatment group	Conventional treatment group	*p* value
Body mass index			
Baseline	24.7 ± 4.4 (n = 149)	24.9 ± 3.6 (n = 153)	0.63
26 weeks (change from baseline)	0.2 ± 1.4 (n = 144)	0.0 ± 1.8 (n = 158)	0.24
52 weeks (change from baseline)	0.1 ± 1.3 (n = 149)	− 0.2 ± 1.6 (n = 156)^a^	0.051
78 weeks (change from baseline)	0.2 ± 1.5 (n = 147)	− 0.2 ± 1.6 (n = 152)^a^	0.062
104 weeks (change from baseline)	0.3 ± 1.9 (n = 146)	− 0.3 ± 1.7 (n = 150)^a^	0.004
156 weeks (change from baseline)	0.0 ± 1.5 (n = 130)	− 0.5 ± 1.9 (n = 136)^a^	0.018
208 weeks (change from baseline)	− 0.1 ± 1.5 (n = 136)	− 0.5 ± 2.0 (n = 136)^a^	0.057
260 weeks (change from baseline)	− 0.2 ± 1.6 (n = 123)	− 0.6 ± 2.0 (n = 130)^a^	0.070
312 weeks (change from baseline)	− 0.3 ± 1.6 (n = 123)^a^	− 0.6 ± 2.1 (n = 122)^a^	0.37
364 weeks (change from baseline)	− 0.4 ± 1.8 (n = 117)^a^	− 0.9 ± 1.9 (n = 118)^a^	0.038
416 weeks (change from baseline)	− 0.7 ± 1.9 (n = 107)^a^	− 1.2 ± 2.0 (n = 106)^a^	0.045
468 weeks (change from baseline)	− 0.7 ± 1.9 (n = 97)^a^	− 1.3 ± 2.2 (n = 153)^a^	0.031
520 weeks (change from baseline)	− 0.8 ± 2.0 (n = 92)^a^	− 1.5 ± 2.3 (n = 91)^a^	0.031
HbA1c at baseline (%)			
Baseline	7.3 ± 0.8 (n = 149)	7.2 ± 0.9 (n = 153)	0.42
26 weeks (change from baseline)	− 0.4 ± 0.7 (n = 149)^a^	0.0 ± 0.9 (n = 153)	< 0.001
52 weeks (change from baseline)	− 0.4 ± 0.6 (n = 148)^a^	− 0.1 ± 0.8 (n = 153)	< 0.001
78 weeks (change from baseline)	− 0.4 ± 0.8 (n = 149)^a^	0.0 ± 1.1 (n = 153)	< 0.001
104 weeks (change from baseline)	− 0.3 ± 0.7 (n = 149)^a^	− 0.1 ± 0.8 (n = 153)	0.006
156 weeks (change from baseline)	− 0.3 ± 0.8 (n = 140)^a^	− 0.1 ± 0.9 (n = 143)	0.23
208 weeks (change from baseline)	− 0.2 ± 1.0 (n = 137)^a^	− 0.2 ± 0.9 (n = 139)^a^	1.00
260 weeks (change from baseline)	− 0.3 ± 0.8 (n = 128)^a^	− 0.2 ± 1.0 (n = 131)	0.31
312 weeks (change from baseline)	− 0.1 ± 1.1 (n = 124)	− 0.2 ± 0.9 (n = 124)^a^	0.49
364 weeks (change from baseline)	− 0.1 ± 1.0 (n = 118)	− 0.1 ± 1.0 (n = 118)	0.97
416 weeks (change from baseline)	− 0.1 ± 0.9 (n = 104)	− 0.1 ± 0.9 (n = 107)	0.96
468 weeks (change from baseline)	0.0 ± 1.2 (n = 99)	− 0.2 ± 1.1 (n = 98)	0.27
520 weeks (change from baseline)	− 0.2 ± 1.1 (n = 93)	− 0.2 ± 1.1 (n = 91)^a^	0.54

Data are mean ± SD. Between-group differences in parameters at baseline were analyzed by Student’s *t*-test. Within-group differences in parameters from baseline to each observation point were analyzed by the one-sample *t*-test. Between-group differences in parameters from baseline to each observation point were analyzed by Student’s *t*-test.

^a^*p* < 0.05.

HbA1c at baseline was 7.3 ± 0.8% in the alogliptin group and 7.2 ± 0.9% in the conventional treatment group, with no significant difference (Table 2). In the alogliptin group, HbA1c was significantly decreased from baseline between 26 and 260 weeks after alogliptin initiation, and gradually returned to near-baseline levels. In the conventional treatment group, the HbA1c decrease from baseline was significant at 208, 312 and 520 weeks. The decrease in HbA1c was larger in the alogliptin group than that in the conventional treatment group from 26 to 104 weeks, and this difference was lost by 156 weeks.

There were no major changes in systolic blood pressure (BP), diastolic BP, total cholesterol levels, high-density lipoprotein cholesterol (HDL) cholesterol levels and triglyceride levels from baseline between the two groups throughout the follow-up period (data not shown). With respect to renal function, estimated glomerular filtration rate (eGFR) was significantly decreased from baseline in both groups throughout the follow-up period, though the changes were not significantly different between the two groups at any time point (Table S2).

### Primary and secondary endpoints

**Figure 1:**
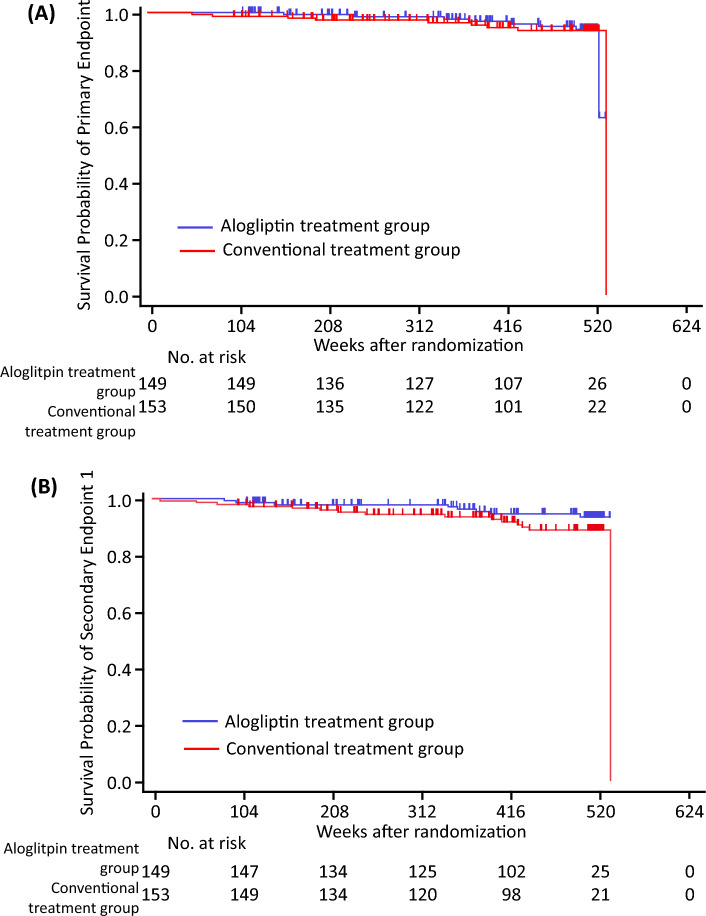
Kaplan–Meier estimates of cardiovascular events. The figures show Kaplan–Meier survival curves. (**A**) Primary endpoint. (**B**) Secondary endpoint 1. Blue lines indicate the alogliptin treatment group, and red lines indicate the conventional treatment group. Primary endpoint: the first occurrence of a major cardiovascular event, which included death from any cause, acute myocardial infarction, and stroke. Endpoint 1: ischemic heart disease (sudden cardiac death, acute myocardial infarction, hospitalization for unstable angina, and coronary revascularization procedure), cerebrovascular events (ischemic stroke, intracerebral hemorrhage, and subarachnoid hemorrhage), and arteriosclerosis obliterans.

During the 520-week follow-up period, the composite cardiovascular events of the primary endpoint occurred in 8 subjects (5.4%) in the alogliptin group and 9 subjects in the conventional treatment group (5.9%) (Table 3 and Table S3). The total number of events was extremely low, and the incidence of the primary endpoint did not differ significantly between the two groups (Table 3 and Fig. 1). In the unadjusted Cox proportional hazards models, higher HbA1c and urinary albumin excretion (UAE) were significantly associated with the incidence of the primary endpoint (Table S4). Multivariate Cox proportional hazards models adjusted for age and gender (model 1), and for age, gender, BMI, HbA1c level, and UAE level (model 2), showed no significant differences in the incidence of the primary endpoint (Table 4).

**Table 3:** Comparison of primary and secondary endpoints.

	Alogliptin treatment group	Conventional treatment group	*p* value	Unadjusted HR (95% CI)
Primary endpoint	8 (5.4)	9 (5.9)	0.86	0.92 (0.34 to 2.46)
Secondary endpoint 1	8 (5.4)	15 (9.8)	0.19	0.56 (0.24 to 1.35)
Secondary endpoint 2	14 (9.4)	17 (11.1)	0.62	0.83 (0.40 to 1.71)
Secondary endpoint 3	2 (1.3)	7 (4.6)	0.15	0.33 (0.07 to 1.62)
Secondary endpoint 4	2 (1.3)	8 (5.2)	0.09	0.28 (0.06 to 1.35)

Data are presented as number (%) of patients. Significance was assessed by the log-rank test.

Primary endpoint: the first occurrence of a major cardiovascular event, which included death from any cause, acute myocardial infarction, and stroke. Endpoint 1: ischemic heart disease (sudden cardiac death, acute myocardial infarction, hospitalization for unstable angina, and coronary revascularization procedure), cerebrovascular events (ischemic stroke, intracerebral hemorrhage, and subarachnoid hemorrhage), and arteriosclerosis obliterans. Endpoint 2: Endpoint 1 plus death due to any cause. Endpoint 3: cardiovascular death, acute myocardial infarction, and stroke. Endpoint 4: Endpoint 3 plus hospitalization for unstable angina.

CI, confidence interval; HR, hazard ratio.

**Table 4:** Multivariate Cox Proportional Hazard Models for Primary and Secondary Endpoints.

	Model 1		Model 2	
	HR (95% CI)	*p* value	HR (95% CI)	*p* value
Primary endpoint	0.88 (0.32 to 2.39)	0.80	0.80 (0.28 to 2.35)	0.69
Secondary endpoint 1	0.55 (0.23 to 1.32)	0.18	0.55 (0.23 to 1.32)	0.18
Secondary endpoint 2	0.79 (0.38 to 1.64)	0.53	0.73 (0.34 to1.59)	0.43
Secondary endpoint 3	0.33 (0.07 to 1.62)	0.17	0.19 (0.03 to 1.09)	0.06
Secondary endpoint 4	0.28 (0.06 to 1.34)	0.11	0.30 (0.06 to 1.44)	0.13

Models were derived from Cox proportional hazards regression analysis. Model 1: adjusted for age and gender. Model 2: adjusted for age, gender, and variables with a p value lower than 0.1 in a univariate Cox proportional hazards model.

Primary endpoint: the first occurrence of a major cardiovascular event, which included death from any cause, acute myocardial infarction, and stroke. Endpoint 1: ischemic heart disease (sudden cardiac death, acute myocardial infarction, hospitalization for unstable angina, and coronary revascularization procedure), cerebrovascular events (ischemic stroke, intracerebral hemorrhage, and subarachnoid hemorrhage), and arteriosclerosis obliterans. Endpoint 2: Endpoint 1 plus death due to any cause. Endpoint 3: cardiovascular death, acute myocardial infarction, and stroke. Endpoint 4: Endpoint 3 plus hospitalization for unstable angina.

HR, hazard ratio.

The composite cardiovascular events of secondary endpoint 1 occurred in 8 subjects (5.4%) in the alogliptin group and 15 subjects in the conventional treatment group (9.8%) (Table 3 and Table S3), with no significant between-group differences in their incidence (Table 3 and Fig. 1). Multivariate Cox proportional hazards models adjusted for age and gender, and for age, gender, BMI, and systolic BP (Table S5), demonstrated no significant differences in the incidence of secondary endpoint 1 (Table 4).

There was no significant difference between the two groups in the incidence of the composite cardiovascular events of secondary endpoint 2 (Table 3 and Fig. 1). Multivariate Cox proportional hazards models adjusted for age and gender, and for age, gender, estimated duration of diabetes, BMI, systolic BP, and UAE (Table S5), showed no significant differences in the incidence of secondary endpoint 2 (Table 4).

Of note, the incidences of the composite cardiovascular events of secondary endpoints 3 and 4 were nonsignificantly lower in the alogliptin group than in the conventional treatment group (Tables 3, 4, and Table S6).

### Post hoc analysis

The study results may have been influenced by the fact that there was a low number of cardiovascular events and the conventional treatment group was characterized by the addition of DPP-4 inhibitors during the follow-up period. Thus, we conducted exploratory post hoc analyses to better understand the effects of DPP-4 inhibitors on cardiovascular events, with a focus on the composite cardiovascular events of the primary endpoint and of secondary endpoints 1 and 2. The post hoc landmark analysis between 104 and 520 weeks showed no significant differences between the treatment groups in the incidence of the composite cardiovascular events of the primary endpoint or of secondary endpoints 1 or 2 (Table S7 and Figure S2).

Next, we assessed whether DPP-4 inhibitors themselves could reduce the risk of cardiovascular events. There was no significant difference between subjects who did or did not receive DPP-4 inhibitors in terms of the incidence of the composite cardiovascular events of each endpoint, although the addition of DPP-4 inhibitors was associated with a lower incidence of the composite cardiovascular events of secondary endpoint 2 (6.3% vs. 12.3%; unadjusted hazard ratio (HR), 0.46 [95% confidence interval (CI) 0.19 to 1.14, *p* = 0.084] (Table S8).

Finally, we performed a recurrent event analysis for each endpoint. There were no significant differences between the alogliptin and conventional groups in the incidence rates of the composite cardiovascular events of the primary endpoint or of secondary endpoint 2, while the incidence rate for secondary endpoint 1 was nonsignificantly lower in the alogliptin group than in the conventional treatment group (7.2 vs. 14.6 per 1000 person-years, *p* = 0.066) (Table 5). On the other hand, the incidence rates for each endpoint were significantly lower in the DPP-4 inhibitor group than in the non-DPP-4 inhibitor group (primary endpoint: 5.8 vs. 13.3 per 1000 person-years, *p* = 0.041; secondary endpoint 1: 8.4 versus 22.2 per 1000 person-years, *p* = 0.004; and secondary endpoint 2: 12.3 versus 24.4 per 1000 person-years, *p* = 0.019, respectively) (Table 5).

**Table 5:** Recurrent event analyses for each endpoint.

Variables	Group	Number of total events	Incidence rate (1000 person-years)	Number of events per patient	*p* value
Primary endpoint	Alogliptin treatment group (n = 149)	8	6.3	0.054 ± 0.226	0.54
	Conventional treatment group (n = 153)	10	8.0	0.065 ± 0.273	
Secondary endpoint 1	Alogliptin treatment group (n = 149)	9	7.2	0.060 ± 0.266	0.066
	Conventional treatment group (n = 153)	18	14.6	0.118 ± 0.396	
Secondary endpoint 2	Alogliptin treatment group (n = 149)	15	15	0.101 ± 0.324	0.24
	Conventional treatment group (n = 153)	21	21	0.137 ± 0.430	
Primary endpoint	DPP-4 inhibitor group (n = 238)	12	5.8	0.050 ± 0.238	0.041
	Non-DPP-4 inhibitor group (n = 64)	6	13.3	0.094 ± 0.294	
Secondary endpoint 1	DPP-4 inhibitor group (n = 237)	17	8.4	0.072 ± 0.290	0.004
	Non-DPP-4 inhibitor group (n = 65)	10	22.2	0.154 ± 0.475	
Secondary endpoint 2	DPP-4 inhibitor group (n = 237)	25	12.3	0.105 ± 0.347	0.019
	Non-DPP-4 inhibitor group (n = 65)	11	24.4	0.169 ± 0.486	

*p* values were calculated using Poisson regression analysis.

Primary endpoint: the first occurrence of a major cardiovascular event, which included death from any cause, acute myocardial infarction, and stroke. Endpoint 1: ischemic heart disease (sudden cardiac death, acute myocardial infarction, hospitalization for unstable angina, and coronary revascularization procedure), cerebrovascular events (ischemic stroke, intracerebral hemorrhage, and subarachnoid hemorrhage), and arteriosclerosis obliterans. Endpoint 2: Endpoint 1 plus death due to any cause. Endpoint 3: cardiovascular death, acute myocardial infarction, and stroke. Endpoint 4: Endpoint 3 plus hospitalization for unstable angina.

DPP-4; dipeptidyl peptidase-4, CI; confidence interval, HR; hazard ratio.

### Safety

Cancer occurred in 11 subjects (7.4%) in the alogliptin group and 17 subjects (11.1%) in the conventional treatment group (Table S9). There were nonsignificant differences between the two groups in terms of the number of patients with each type of cancer. There was no significant difference between the two groups in the overall incidence of cancer (unadjusted HR 0.62; 95% CI 0.29 to 1.33; *p* for log-rank test = 0.22). In unadjusted Cox proportional hazards models, older age, longer estimated duration of diabetes, and the use of angiotensin-converting enzyme inhibitors (ACEs) or angiotensin II receptor blockers (ARBs) were significantly associated with the incidence of cancer (Table S10). Multivariate Cox proportional hazards models adjusted for age, gender, estimated duration of diabetes, the use of ACEs or ARBs, and the use of metformin showed no significant difference in cancer incidence between the alogliptin and conventional treatment group (0.64; 95% CI 0.30 to 1.36; *p* = 0.24).

Very few subjects experienced hypoglycemia during the follow-up period. There were no significant differences between the two groups in the incidence of confirmed, protocol-defined, or severe hypoglycemic events (Table S11).

## Discussion

In this study, the incidence of coronary heart disease and stroke were relatively low, at 5.6 and 2.4 per 1000 person-years, respectively. These values are similar to those demonstrated by previous cohort studies conducted in Japan^20–22^. Specifically, the incidences of coronary heart disease in Japanese people with type 2 diabetes in the Hisayama study^20^, Japan Diabetic Complication Study (JDCS)^21^, and Japan Diabetes Clinical Data Management (JDDM) study^22^ were 5.0, 8.3, and 4.4 per 1000 person-years, while those of stroke were 6.5, 7.6, and 3.1 per 1000 person-years, respectively. These rates are lower than those found by the General Practice Research Database study conducted in the United Kingdom, which reported that the incidences of cardiovascular events and cerebrovascular events were 18.3 and 11.9 per 1000 person-years, respectively, in people with type 2 diabetes^23,24^. In this study, the physician in charge provided optimal comprehensive treatment throughout the study period, including taking into account the treatment targets in the guideline. As a result, participants in both groups had fairly good control of glycemic levels, BP, and lipids during the study period. Accordingly, the relatively high rates at which optimal, comprehensive management was achieved may have accounted for the lower incidence of cardiovascular events.

The low incidence of cardiovascular events in this study made it difficult to determine the impact of early alogliptin treatment on cardiovascular events during the 104-week period of the randomized trial. There were no significant differences in the primary or secondary composite cardiovascular endpoints between the two groups, although alogliptin treatment was associated with a somewhat reduced incidence of the composite cardiovascular events of several secondary endpoints. Similarly, no significant differences between the two groups were observed in the post hoc landmark analysis or recurrent events analysis. However, it should be noted that alogliptin treatment during the 104-week period of the randomized trial was associated with about a 49% reduction in the incidence of the composite cardiovascular events of secondary endpoint 1. Further, in the recurrent event analysis, the rate of composite cardiovascular events per 1000 person-years, defined as the same events as the secondary endpoint 1, was numerically but nonsignificantly lower in the alogliptin group than in the conventional treatment group (*p* = 0.066). Further long-term studies with larger numbers of participants are needed to determine whether alogliptin treatment can reduce the incidence of cardiovascular events.

We previously reported that alogliptin treatment slowed the progression of carotid atherosclerosis^25^. In addition, preclinical studies have suggested that DPP-4 inhibitors may have beneficial effects on atherosclerosis, through both GLP-1-dependent and -independent mechanisms^26–28^. Indeed, subjects who received DPP-4 inhibitors before the onset of the first cardiovascular event were about 40–60% less likely to develop the composite cardiovascular events of the primary endpoint and those of secondary endpoints 1 and 2 compared to those who did not, although the differences were not statistically significant. Furthermore, recurrent events analysis showed that the subjects with early DPP-4 inhibitor treatment had significantly lower rates of composite cardiovascular events per 1000 person-years compared to their counterparts (Table 4). Accordingly, DPP-4 inhibitors have the potential to reduce the risk of cardiovascular events.

Previous CVOTs have not shown that DPP-4 inhibitors have any significant prognostic superiority over placebo in people with type 2 diabetes and a history of CVD or a high risk of CVD^2–5^. Since these safety outcome studies were originally designed as non-inferiority clinical trials, they probably lacked sufficient power to evaluate the beneficial effects of DPP-4 inhibitors on CVD. In general, the required number of cardiovascular events in a superiority trial is larger than that in a non-inferiority trial^29^. On the other hand, a nationwide cohort study using claims data from the National Health Insurance in Taiwan showed that DPP-4 inhibitors had a stronger cardioprotective effect in people with type 2 diabetes and no apparent history of CVD^30^. Interestingly, a previous study demonstrated greater glucose-lowering effects of DPP4 inhibitors in Asians compared to non-Asians, suggesting that the efficacy of DPP4 inhibitors may differ between populations^16^. In particular, differences in BMI between ethnic groups may contribute to differences in the glucose-lowering effects of DPP-4 inhibitors. We could not rule out the possibility that several factors, such as CVD history, race, and the presence or absence of obesity, differentially impact the effect of DPP-4 inhibitors on CVD. In addition, a recent retrospective analysis demonstrated that DPP-4 inhibitors reduced long-term cardiovascular risk in Japanese people with mean BMI of 24.7 kg/m^2^ despite a population who underwent percutaneous coronary intervention for coronary artery disease^31^. This data suggests that DPP-4 inhibitors may reduce the risk of cardiovascular events even in people with a history of CVD if they were Asia and were not too obese. Therefore, a larger-scale prospective clinical trial with a longer observation period is required to assess the cardiovascular efficacy of DPP-4 inhibitors in a wider range of people with type 2 diabetes.

Several preclinical studies have demonstrated that DPP-4 inhibitors have anti-inflammatory and profibrotic effects, and even anti-tumor effects in various organs^32,33^. On the other hand, there are concerns that DPP-4 inhibitors may increase the risk of pancreatic cancer due to pancreatic expansion^34^. Nevertheless, previous studies have shown conflicting results regarding the correlation between the use of DPP4 inhibitors and the incidences of various types of cancer, including pancreatic cancers^34–36^. In this study, early alogliptin treatment did not increase cancer risk compared to conventional treatment, although the overall cancer incidence rate during the observation period was low. Further studies such as meta-analyses, long-term studies, and real-world studies are needed to determine if DPP-4 inhibitors affect the incidence of cancer.

Treatment-induced hypoglycemia is regarded as a major concern in the management of type 2 diabetes. Consistent with their mechanism of action, DPP-4 inhibitors used as monotherapy are associated with a low risk of hypoglycemic episodes^37^. In this study, there was a low incidence of hypoglycemia, as in a previous study^19^, and the incidence did not differ statistically between the two groups. The low incidence in both groups is likely due to the fact that the doses of other glucose-lowering agents were allowed to be adjusted during the study period.

The present study has certain limitations. First, this was a prospective, observational cohort study with no medication restrictions in either group. Second, the small sample size and event incidence in this study may have made it difficult to assess differences in cardiovascular events between groups. Third, we only recruited Japanese people with type 2 diabetes and without a history of cardiovascular events. These constraints may have limited the generalizability of our results. Finally, it remains inconclusive whether DPP-4 inhibitors have impacts on the risk of heart failure^2–5,38^. It would be interesting to determine whether early alogliptin initiation impacts long-term outcome of heart failure in this study. However, heart failure related outcomes were not included in this study.

## Conclusions

The early initiation of alogliptin was not associated with a reduced risk of composite cardiovascular disease, which may be partly attributed to the small number of events and/or the addition of DPP-4 inhibitors during follow-up period. A large-scale prospective trial is required to establish the usefulness of DPP-4 inhibitors for primary prevention of CVD in people with type 2 diabetes.

## Methods

### Study design

This prospective, observational cohort study was designed to determine whether early alogliptin administration improved cardiovascular outcomes during a 520-week follow-up period. The original SPEAD-A trial was a multicenter, prospective, randomized, open-label, blinded-endpoint study, as described previously^25,39^. Briefly, Japanese people with type 2 diabetes and no apparent history of CVD who periodically attended the Outpatient Diabetes Clinics at 11 centers across Japan were asked to participate. A total of 341 subjects were randomly allocated to either the alogliptin group (n = 172) or the conventional treatment group (n = 169) between March 2011 and June 2013. Among them, 304 subjects completed the follow-up. After the completion of follow-up in the SPEAD-A trial (between May 2013 and March 2014), each subject was asked to participate in this SPEAD-A Extension study. Two subjects did not give consent, and therefore 302 subjects were enrolled. During the SPEAD-A Extension study, subjects received routine diabetes care through their usual healthcare providers and there was no restriction in medication use, including DPP-4 inhibitors, in either the alogliptin or convention group. At study visits during the SPEAD-A Extension study, clinical outcomes and adverse events were ascertained and adjudicated by each investigator in an open fashion, and clinical and biochemical data were collected at the following numbers of weeks after randomization: 104 (starting point of this study), 156, 208, 260, 312, 364, 416, 468, and 520. The protocol was approved by the Institutional Review Board at each participating institution (Juntendo University Graduate School of Medicine, Osaka University Graduate School of Medicine, Juntendo Tokyo Koto Geriatric Medical Center, Naka Kinen Clinic, Osaka Police Hospital, Osaka General Medical Center, Kansai Rosai Hospital, Jiyugaoka Medical Clinic), and the study was conducted in compliance with the Declaration of Helsinki and current legal regulations in Japan. This study was registered in the University Hospital Medical Information Network Clinical Trials Registry (UMIN-CTR), which is a non-profit organization in Japan that meets the requirements of the International Committee of Medical Journal Editors (ICMJE) (UMIN000010534).

### Study outcomes

The primary study outcome was the first occurrence of a major cardiovascular event, which included death from any cause, acute myocardial infarction, and stroke, in each case verified by hospital records. Definitions of the endpoints are provided in the supplementary Materials.

The secondary outcomes were the first occurrences of the major cardiovascular events described below.

1. Endpoint 1: ischemic heart disease (sudden cardiac death, acute myocardial infarction, hospitalization for unstable angina, and coronary revascularization procedure), cerebrovascular events (ischemic stroke, intracerebral hemorrhage, and subarachnoid hemorrhage), and arteriosclerosis obliterans.
2. Endpoint 2: 1) plus death due to any cause.
3. Endpoint 3: cardiovascular death, acute myocardial infarction, and stroke.
4. Endpoint 4: 3) plus hospitalization for unstable angina.
5. Endpoint 5: new onset-cancer as a safety endpoint.

The other outcomes were changes in HbA1c, eGFR, and lipid parameters (total cholesterol, HDL cholesterol, triglycerides), and the appearance of any adverse event.

### Biochemical tests

Blood samples were obtained after overnight fasting, as described previously^25,39^. Serum lipids, HbA1c (National Glycohemoglobin Standardization Program), glucose, and creatinine were measured with standard techniques. UAE was measured by the improved bromocresol purple method using a spot urine sample. The eGFR was calculated using the following formula: eGFR (ml/min per 1.73 m^2^) = 194 × Age^−0.287^ × serum creatinine^−0.1094^ (× 0.739 for females)^40^.

### Statistical analysis

The analysis set was the intent-to-treat (ITT) population who had completed the SPEAD-A study and had given consent to participate in the Extension study. Results are presented as mean ± standard deviation (SD) or median (quartiles 1 and 3) for continuous variables, and as number (proportion) of subjects for categorical variables. Baseline and follow-up group comparisons were assessed with Student’s t-test or Wilcoxon’s rank sum test for continuous variables, and with Fisher’s exact test for categorical variables. Changes from baseline to treatment visits within groups were assessed with the one-sample t-test and Wilcoxon’s signed-rank test. The number and percentage of subjects reporting hypoglycemia is presented by treatment group, with between-group differences compared using Fisher’s exact test.

The times to the events defined by the primary and secondary endpoints were estimated using the Kaplan–Meier method and compared between treatment groups with the log-rank test. To evaluate the effect of alogliptin treatment on the primary and secondary endpoints, univariate and multivariate Cox proportional hazards models were used to estimate the HR and its 95% CI. Conventional risk factors evaluated with clinical, biochemical, or metabolic testing were included in the models based on clinical judgment. Model 1 included age and gender as factors. Model 2 included model 1 adjustments plus adjustment for factors with a P value lower than 0.1 in a univariate Cox proportional hazards model.

We conducted post hoc analyses to better understand the effects of alogliptin or DPP-4 inhibitors on cardiovascular events. First, we conducted a landmark analysis at 104 weeks to determine whether 104 weeks of alogliptin treatment could reduce the risk of cardiovascular events after the randomized trial period. Subjects who had developed cardiovascular events were excluded at 104 weeks. Next, to determine whether DPP-4 inhibitors alone could reduce the risk of cardiovascular events, subjects were divided into two groups: those who received DPP-4 inhibitors before the onset of cardiovascular events (DPP-4 inhibitor groups) and those who did not (non-DPP-4 inhibitor group). Finally, the number of events described in the primary and secondary outcomes were analyzed using a Poisson regression model to compare the recurrent incidence rates between treatment groups. The model included the treatment group as a factor and the follow-up period as an offset term. The incident rate (1000 person-years) of each endpoint was calculated as 1000 × the total number of CVD event(s) / the cumulative number of follow-up years.

All statistical tests were two-sided, with a 5% significance level. All analyses were performed using SAS software version 9.4 (SAS Institute, Cary, NC). The administrative office of the SPEAD-A trial analyzed the data based on instructions from an independent biostatistician.

### Ethics declarations

The protocol was approved by the institutional review board of each participating institution in compliance with the Declaration of Helsinki and current legal regulations in Japan. Written informed consent was obtained from all participants after a full explanation of the study.

### Supplementary Information

Supplementary Information.

## Data Availability

The analyzed datasets are available from the corresponding author on reasonable request.
