# Supplementary material for: Long-term efficacy and safety of early alogliptin initiation in subjects with type 2 diabetes: an extension of the SPEAD-A study
Source: Sci Rep. 2023 Sep 5;13:14649. doi: 10.1038/s41598-023-41036-1 (PMC10480471; doi:10.1038/s41598-023-41036-1)
Supplement: Supplementary file 1 — Supplementary Information. [file 41598_2023_41036_MOESM1_ESM.pdf]

SUPPLEMENTARY MATERIAL

Long-term efficacy and safety of early alogliptin initiation in subjects with type 2 diabetes: an extension of the SPEAD-A study

Tomoya Mita,
Naoto Katakami,
Hidenori Yoshii,
Tomio Onuma,
Hideaki Kaneto,
Takeshi Osonoi,
Toshihiko Shiraiwa,
Tetsuyuki Yasuda,
Yutaka Umayahara,
Tsunehiko Yamamoto,
Hiroki Yokoyama,
Nobuichi Kuribayashi,
Hideaki Jinnouchi,
Masahiko Gosho,
Iichiro Shimomura,
Hirotaka Watada,

Collaborators on the Study of Preventive Effects of Alogliptin on Diabetic Atherosclerosis (SPEAD-A) Trial

Definitions of cardiovascular events.

1. Definition of ischemic heart disease

1.1. Cardiovascular death

Cardiovascular death includes death within 24 hours of symptom onset with no clear cause other than CVD.

1.2. Myocardial infarction

Diagnosis of myocardial infarction was required to meet at least two of the following three criteria: clinical symptoms, cardiac biomarker information, and electrocardiographic findings.

1) Clinical presentation (at least one)

· Chest pain lasting more than 30 minutes.

· Pulmonary edema.

· Cardiogenic shock without evidence of another cause.

2) Cardiac biomarker elevations (elevation of any of the following myocardium-derived markers)

· Serum creatine phosphokinase or creatine phosphokinase-MB isoenzyme level greater than twice the upper limit of normal.

· Positive rapid test for troponin-T or heart-type fatty acid-binding protein.

3) Electrocardiographic findings and coronary angiography (findings of myocardial infarction) (at least one)

· Development of new pathological Q waves and new ST elevation in more than 2 leads

· New left bundle branch block or new ST-segment/T-wave changes.

· Identification of coronary stenosis resulting in ≥50% diameter reduction in any major epicardial vessel or occlusion by angiography.

1.3. Unstable angina pectoris

Of the following four items, 1) and 2) are required, and either 3) or 4) must be met.

1) Chest pain.

Chest pain is defined by one or more of the following signs.

a. Ischemic discomfort for ≥20 minutes.

b. New-onset (<2 months) severe angina (Canadian Cardiovascular Society (CCS) Grading Scale classification severity III or IV

c. Worsening angina (intensity, duration, and/or frequency) with an increase in severity of at least 1 CCS class to at least CCS class III

2) Confirmation of coronary artery lesion responsible for acute coronary syndrome by imaging (coronary angiography or multi-detector row CT).

3) New ST-segment/T-wave changes by electrocardiogram.

4) New regional wall motion abnormality identified by echocardiography.

1.4. Coronary revascularization

·Percutaneous coronary angioplasty or coronary artery bypass

2. Definition of cerebrovascular disease.

· Cerebrovascular disease was defined as an acute episode of neurological dysfunction caused by focal or global brain vascular injury and was diagnosed by neurologists as follows.

2.1 Cerebral infarction

· Cerebral infarcts consistent with clinical findings were confirmed by CT, MRI, or autopsy; diffusion-weighted magnetic resonance imaging was necessary to confirm cerebral infarcts.

2.2. Cerebral hemorrhage

· Cerebral hemorrhages were confirmed by CT, MRI, or autopsy. However, hemorrhagic infarction and intratumoral hemorrhages are not classified as cerebral hemorrhage; hemorrhagic infarction is classified as cerebral infarction.

2.3. Subarachnoid hemorrhage

· Bleeding in the subarachnoid space was confirmed by CT or MRI. The presence of blood in spinal fluid was confirmed by lumbar puncture.

3 Peripheral arterial disease

· Peripheral arterial disease was defined as intermittent claudication and a resting ankle brachial index <0.9, and/or CT or MRI imaging evidence of the presumed culprit lesion(s). Or peripheral arterial revascularization was performed.

Table S1. Glucose-lowering agent use.

Parameters	Alogliptin treatment group	Conventional treatment group	P value
Glucose-lowering agents	149(100)	133(87)	<0.001
Baseline	148(99)	147(96)	0.12
26 weeks	148(99)	148(97)	0.21
52 weeks	148(99)	147(96)	0.12
78 weeks	148(99)	146(95)	0.067
104 weeks	143(99)	141(97)	0.45
156 weeks	134(98)	138(98)	1.00
208 weeks	131(99)	135(97)	0.37
260 weeks	125(99)	126(98)	0.62
312 weeks	121(99)	118(99)	1.00
364 weeks	112(99)	113(99)	1.00
416 weeks	104(100)	103(100)	-
468 weeks	97(100)	94(100)	-
Alogliptin			
Baseline	149 (100)	0 (0)	<0.001
26 weeks	144 (97)	0 (0)	<0.001
52 weeks	143 (96)	0 (0)	<0.001
78 weeks	143 (96)	0 (0)	<0.001
104 weeks	143 (96)	1 (1)	<0.001
156 weeks	128 (88)	9 (6)	<0.001
208 weeks	111 (81)	16 (11)	<0.001
260 weeks	104 (79)	19 (14)	<0.001
312 weeks	91 (72)	19 (15)	<0.001
364 weeks	82 (67)	24 (20)	<0.001
416 weeks	72 (64)	20 (18)	<0.001
468 weeks	66 (63)	19 (18)	<0.001
520 weeks	58 (60)	19 (20)	<0.001
DPP-4 inhibitors			
Baseline	149 (100)	0 (0)	<0.001
26 weeks	144 (97)	0 (0)	<0.001
52 weeks	143 (96)	2 (1)	<0.001
78 weeks	143 (96)	4 (3)	<0.001
104 weeks	143 (96)	3 (2)	<0.001
156 weeks	137 (94)	36 (25)	<0.001

208 weeks	126 (92)	50 (35)	<0.001
260 weeks	123 (93)	57 (41)	<0.001
312 weeks	117 (93)	66 (51)	<0.001
364 weeks	113 (93)	71 (60)	<0.001
416 weeks	101 (89)	68 (60)	<0.001
468 weeks	91 (88)	61 (59)	<0.001
520 weeks	80 (82)	59 (63)	0.003
Metformin			
Baseline	77(52)	73(48)	0.57
26 weeks	75(50)	76(50)	1.00
52 weeks	79(53)	77(50)	0.65
78 weeks	80(54)	79(52)	0.73
104 weeks	81(54)	81(53)	0.82
156 weeks	75(52)	86(59)	0.24
208 weeks	72(53)	83(59)	0.33
260 weeks	68(52)	81(58)	0.27
312 weeks	71(56)	78(60)	0.53
364 weeks	71(58)	78(66)	0.29
416 weeks	67(59)	79(69)	0.13
468 weeks	62(60)	75(73)	0.056
520 weeks	58(60)	65(69)	0.23
Sulfonylureas			
Baseline	74(50)	88(58)	0.20
26 weeks	76(51)	88(58)	0.30
52 weeks	73(49)	88(58)	0.17
78 weeks	75(50)	86(56)	0.36
104 weeks	74(50)	82(54)	0.56
156 weeks	67(46)	72(49)	0.64
208 weeks	58(42)	63(45)	0.72
260 weeks	53(40)	55(40)	1.00
312 weeks	45(36)	47(36)	1.00
364 weeks	38(31)	48(40)	0.14
416 weeks	31(27)	41(36)	0.20
468 weeks	24(23)	36(35)	0.067
520 weeks	22(23)	32(34)	0.11
Glinides			
Baseline	8(5)	15(10)	0.19

26 weeks	7(5)	17(11)	0.054
52 weeks	7(5)	18(12)	0.035
78 weeks	7(5)	20(13)	0.014
104 weeks	8(5)	22(14)	0.012
156 weeks	4(3)	17(12)	0.005
208 weeks	7(5)	17(12)	0.053
260 weeks	5(4)	15(11)	0.035
312 weeks	6(5)	16(12)	0.043
364 weeks	8(7)	14(12)	0.18
416 weeks	8(7)	16(14)	0.13
468 weeks	14(13)	18(17)	0.45
520 weeks	11(11)	15(16)	0.40
Thiazolidinediones			
Baseline	34(23)	37(24)	0.79
26 weeks	33(22)	37(24)	0.69
52 weeks	34(23)	37(24)	0.79
78 weeks	34(23)	40(26)	0.51
104 weeks	33(22)	41(27)	0.35
156 weeks	29(20)	36(25)	0.40
208 weeks	28(20)	35(25)	0.39
260 weeks	28(21)	33(24)	0.66
312 weeks	25(20)	29(22)	0.65
364 weeks	24(20)	24(20)	1.00
416 weeks	19(17)	22(19)	0.73
468 weeks	18(17)	17(17)	1.00
520 weeks	16(16)	16(17)	1.00
α-glucosidase inhibitors			
Baseline	51(34)	47(31)	0.54
26 weeks	49(33)	63(41)	0.15
52 weeks	48(32)	66(43)	0.058
78 weeks	49(33)	68(44)	0.045
104 weeks	49(33)	69(45)	0.034
156 weeks	42(29)	60(41)	0.037
208 weeks	41(30)	55(39)	0.13
260 weeks	38(29)	53(38)	0.12
312 weeks	31(25)	48(37)	0.031
364 weeks	29(24)	45(38)	0.025

416 weeks	27(24)	40(35)	0.081
468 weeks	25(24)	37(36)	0.070
520 weeks	24(25)	32(34)	0.20
SGLT-2 inhibitors			
Baseline	0(0)	0(0)	-
26 weeks	0(0)	0(0)	-
52 weeks	0(0)	0(0)	-
78 weeks	0(0)	0(0)	-
104 weeks	0(0)	0(0)	-
156 weeks	0(0)	1(1)	1.00
208 weeks	7(5)	3(2)	0.21
260 weeks	17(13)	12(9)	0.33
312 weeks	20(16)	18(14)	0.73
364 weeks	21(17)	24(20)	0.62
416 weeks	28(25)	29(25)	1.00
468 weeks	27(26)	28(27)	0.88
520 weeks	28(29)	31(33)	0.64
Insulin			
Baseline	0(0)	0(0)	-
26 weeks	0(0)	0(0)	-
52 weeks	1(1)	1(1)	1.00
78 weeks	1(1)	2(1)	1.00
104 weeks	1(1)	2(1)	1.00
156 weeks	4(3)	4(3)	1.00
208 weeks	5(4)	4(3)	0.75
260 weeks	5(4)	5(4)	1.00
312 weeks	9(7)	6(5)	0.44
364 weeks	11(9)	3(3)	0.051
416 weeks	14(12)	6(5)	0.065
468 weeks	14(13)	9(9)	0.38
520 weeks	14(14)	12(13)	0.83

Data are number (%) of patients. The two treatment groups were compared by Fisher's exact test.

Table S2. Changes in renal function during the 520-week follow-up period.

Parameters	Alogliptin treatment group	Conventional treatment group	P value
eGFR (mL/min/1.73 m^2)			
Baseline	78±20 (n=149)	77±18 (n=153)	0.50
26 weeks (change from baseline)	-1±10 (n=147)	1±9 (n=153)	0.02
52 weeks (change from baseline)	-1±10 (n=149)	0±11 (n=153)	0.19
78 weeks (change from baseline)	-1±10 (n=149)	0±11 (n=153)	0.43
104 weeks (change from baseline)	-1±10 (n=149)	0±10 (n=153)	0.40
156 weeks (change from baseline)	-3±11 (n=135) [a]	-2±11 (n=137) [a]	0.26
208 weeks (change from baseline)	-4±12 (n=134) [a]	-3±13 (n=132) [a]	0.48
260 weeks (change from baseline)	-7±12 (n=125) [a]	-5±12 (n=127) [a]	0.10
312 weeks (change from baseline)	-9±12 (n=116) [a]	-8±13 (n=119) [a]	0.55
364 weeks (change from baseline)	-13±12 (n=116) [a]	-12±13 (n=111) [a]	0.49
416 weeks (change from baseline)	-15±12 (n=100) [a]	-14±14 (n=103) [a]	0.68
468 weeks (change from baseline)	-14±13 (n=95) [a]	-12±39 (n=94) [a]	0.65
520 weeks (change from baseline)	-17±13 (n=90) [a]	-17±17 (n=87) [a]	0.98

Data are mean ± SD or median (range). Between-group differences in parameters at baseline were analyzed by Student's t-test or Wilcoxon's rank sum test. Within-group differences in parameters from baseline to each observation point were analyzed by the one-sample t-test or Wilcoxon's signed-rank test. Between-group differences in parameters from baseline to each observation point were analyzed by Student's t-test or Wilcoxon's rank sum test. [a]$p<0.05$.

Table S3. Details of cardiovascular endpoints.

Parameters	Alogliptin treatment group	Conventional treatment group
Death from any cause	6	3
Cardiovascular death	0	0
Myocardial infarction	0	3
Unstable angina pectoris	0	1
Coronary revascularization	5	3
Cerebral infarction	2	4
Cerebral hemorrhage	0	1
Subarachnoid hemorrhage	0	1
Peripheral arterial disease	2	2

Data are number of patients.

Table S4. Univariate Cox Proportional Hazard Models for Primary Endpoint.

	HR (95% CI)	P value
Age (years)	1.04(0.98 to 1.10)	0.20
Gender (female)	0.90(0.33 to 2.44)	0.83
BMI (kg/m^2)	0.87(0.75 to 1.01)	0.070
Estimated duration of diabetes (years)	1.03(0.97 to 1.09)	0.33
Current smoker (yes)	1.11(0.31 to 3.92)	0.88
HbA1c (%)	1.55(1.09 to 2.20)	0.015
Systolic BP (mmHg)	1.01(0.98 to 1.04)	0.49
Total cholesterol (mmol/l)	1.00(0.98 to 1.02)	0.89
HDL cholesterol (mmol/l)	0.99(0.96 to 1.03)	0.74
Logarithm of triglyceride levels	1.12(0.44 to 2.88)	0.81
eGFR (mL/min/1.73 m^2)	0.99(0.96 to 1.02)	0.40
Uric acid (µmol/l)	0.80(0.53 to 1.20)	0.28
Logarithm of the UAE value	1.54(1.17 to 2.03)	0.002
Metformin (yes)	1.01(0.38 to 2.70)	0.98
Thiazolidinediones (yes)	1.34(0.38 to 4.74)	0.65
ACEs and/or ARBs (yes)	0.73(0.26 to 2.00)	0.54
Statins (yes)	1.80(0.62 to 5.26)	0.28
Antiplatelet agents (yes)	0.69(0.20 to 2.43)	0.56

ACE, angiotensin-converting enzyme inhibitor; ARB, angiotensin II receptor blocker; BMI, body mass index; BP, blood pressure; CI, confidence interval; eGFR, estimated glomerular filtration rate; HDL, high-density lipoprotein cholesterol; HR, hazard ratio; UAE, urinary albumin excretion.

Table S5. Univariate Cox Proportional Hazard Models for Secondary Endpoints 1 and 2.

	HR (95% CI) for secondary endpoint 1	P value	HR (95% CI) for secondary endpoint 2	P value
Age (years)	1.04(0.99 to 1.10)	0.093	1.06(1.01 to 1.11)	0.011
Gender (female)	1.70(0.67 to 4.35)	0.27	1.35(0.63 to 2.91)	0.44
BMI (kg/m^2)	0.89(0.78 to 1.01)	0.061	0.90(0.81 to 1.00)	0.048
Estimated duration of diabetes (years)	1.03(0.98 to 1.08)	0.29	1.04(0.99 to 1.08)	0.089
Current smoker (yes)	1.78(0.53 to 6.02)	0.35	1.34(0.51 to 3.52)	0.55
HbA1c (%)	1.15(0.74 to 1.78)	0.53	1.23(0.86 to 1.74)	0.25
Systolic BP (mmHg)	1.02(1.00 to 1.05)	0.042	1.02(1.00 to 1.04)	0.080
Total cholesterol (mmol/l)	1.00(0.99 to 1.02)	0.50	1.00(0.99 to 1.01)	0.91
HDL cholesterol (mmol/l)	1.00(0.98 to 1.03)	0.80	1.00(0.97 to 1.02)	0.93
Logarithm of triglyceride levels	0.87(0.37 to 2.06)	0.75	0.95(0.47 to 1.94)	0.89
eGFR (mL/min/ 1.73 m^2)	0.99(0.97 to 1.01)	0.35	0.98(0.96 to 1.00)	0.10
Uric acid (μmol/l)	1.13(0.79 to 1.60)	0.51	1.14(0.84 to 1.54)	0.39
Logarithm of UAE values	1.05(0.78 to 1.42)	0.75	1.25(1.00 to 1.56)	0.046
Metformin (yes)	1.01(0.44 to 2.33)	0.98	1.15(0.56 to 2.37)	0.69
Thiazolidinediones (yes)	1.12(0.41 to 3.05)	0.82	1.04(0.45 to 2.45)	0.92
ACEs and/or ARBs (yes)	0.68(0.29 to 1.58)	0.37	0.58(0.28 to 1.21)	0.15
Statins (yes)	1.60(0.65 to 3.91)	0.31	1.56(0.73 to 3.36)	0.25
Antiplatelet agents (yes)	0.49(0.18 to 1.34)	0.17	0.49(0.21 to 1.15)	0.10

ACE, angiotensin-converting enzyme inhibitor; ARB, angiotensin II receptor blocker; BMI, body mass index; BP, blood pressure; CI, confidence interval; eGFR, estimated glomerular filtration rate; HDL, high-density lipoprotein cholesterol; HR, hazard ratio; UAE, urinary albumin excretion.

Table S6. Univariate Cox Proportional Hazard Models for Secondary Endpoints 3 and 4.

	HR (95% CI) for secondary endpoint 3	P value	HR (95% CI) for secondary endpoint 4	P value
Age (years)	0.99(0.92 to 1.06)	0.77	0.99(0.93 to 1.07)	0.88
Gender (female)	1.05(0.25 to 4.41)	0.94	1.27(0.32 to 5.07)	0.74
BMI (kg/m^2)	0.81(0.64 to 1.01)	0.064	0.84(0.68 to 1.03)	0.095
Estimated duration of diabetes (years)	0.99(0.90 to 1.09)	0.87	0.99(0.90 to 1.08)	0.81
Current smoker (yes)	1.96(0.24 to 15.96)	0.53	2.23(0.28 to 17.85)	0.45
HbA1c (%)	1.64(1.05 to 2.56)	0.028	1.57(1.00 to 2.45)	0.049
Systolic BP (mmHg)	1.02(0.98 to 1.06)	0.34	1.03(1.00 to 1.07)	0.080
Total cholesterol (mmol/l)	1.01(0.98 to 1.03)	0.50	1.01(0.99 to 1.03)	0.46
HDL cholesterol (mmol/l)	1.00(0.96 to 1.05)	0.88	0.99(0.95 to 1.04)	0.83
Logarithm of triglyceride levels	1.06(0.26 to 4.37)	0.94	1.16(0.31 to 4.38)	0.82
eGFR (mL/min/ 1.73 m^2)	1.01(0.97 to 1.04)	0.73	1.00(0.97 to 1.04)	0.80
Uric acid (μmol/l)	0.53(0.27 to 1.01)	0.052	0.65(0.37 to 1.16)	0.15
Logarithm of UAE values	1.34(0.87 to 2.08)	0.18	1.37(0.91 to 2.05)	0.13
Metformin (yes)	0.61(0.15 to 2.55)	0.50	0.51(0.13 to 2.03)	0.34
Thiazolidinediones (yes)	2.31(0.28 to 18.80)	0.43	2.65(0.33 to 21.20)	0.36
ACEs and/or ARBs (yes)	1.37(0.33 to 5.74)	0.67	1.02(0.27 to 3.82)	0.97
Statins (yes)	2.24(0.45 to 11.09)	0.32	2.60(0.54 to 12.54)	0.23
Antiplatelet agents (yes)	1.05(0.13 to 8.55)	0.96	1.19(0.15 to 9.51)	0.87

ACE, angiotensin-converting enzyme inhibitor; ARB, angiotensin II receptor blocker; BMI, body mass index; BP, blood pressure; CI, confidence interval; eGFR, estimated glomerular filtration rate; HDL, high-density lipoprotein cholesterol; HR, hazard ratio; UAE, urinary albumin excretion.

Table S7. Comparison of Primary and Secondary Endpoints after the landmark time.

	Alogliptin treatment group	Conventional treatment group	P for log-rank test	Unadjusted HR (95% CI)
Primary endpoint	8(5.4)	7(4.6)	0.89	1.08(0.39,2.98)
Secondary endpoint 1	6(4.0)	12(7.8)	0.15	0.49(0.18,1.31)
Secondary endpoint 2	12(8.1)	14(9.2)	0.61	0.82(0.38,1.77)

Data are presented as number (%) of patients. Significance was assessed by the log-rank test.
CI; confidence interval, HR; hazard ratio.

Table S8. Comparison of Primary and Secondary Endpoints in the DPP-4 Inhibitor Group and Non-DPP-4 Inhibitor Group.

	DPP-4 inhibitor group	Non DPP-4 inhibitor group	P for log-rank test	Unadjusted HR (95% CI)
Primary endpoint	11(4.6)	6(9.4)	0.15	0.46(0.16,1.34)
Secondary endpoint 1	15(6.3)	8(12.3)	0.084	0.46(0.19,1.14)
Secondary endpoint 2	22(9.3)	9(13.8)	0.20	0.59(0.26,1.33)

Data are presented as number (%) of patients. Significance was assessed by the log-rank test.
DPP-4; dipeptidyl peptidase-4, CI; confidence interval, HR; hazard ratio.

Table S9. Details of cancers that developed during the study.

Parameters	Alogliptin treatment group	Conventional treatment group
Thyroid cancer	0	2
Lung cancer	2	3
Pharyngeal cancer	1	0
Esophageal cancer	1	0
Gastric cancer	0	1
Colon cancer	1	3
Hepatocellular carcinoma	1	0
Gallbladder cancer	1	0
Neuroendocrine carcinoma of the gallbladder	0	1
Pancreatic cancer	1	0
Intraductal papillary mucinous carcinoma	0	1
Kidney cancer	1	0
Prostate cancer	1	5
Malignant lymphoma	1	1

Data are number of patients.

Table S10. Univariate Cox Proportional Hazard Models for Secondary Endpoint 5 (Cancer).

	HR (95% CI)	P value
Age (years)	1.05(1.00,1.10)	0.030
Gender (female)	1.36(0.61,3.00)	0.45
BMI (kg/m^2)	1.02(0.93,1.12)	0.67
Estimated duration of diabetes (years)	1.05(1.01,1.09)	0.022
Current smoker (yes)	1.00(0.40,2.46)	1.00
HbA1c (%)	0.86(0.53,1.39)	0.54
Systolic BP (mmHg)	0.98(0.96,1.01)	0.26
Total cholesterol (mmol/l)	1.00(0.99,1.02)	0.54
HDL cholesterol (mmol/l)	1.00(0.98,1.02)	1.00
Logarithm of triglyceride levels	0.99(0.47,2.11)	0.98
eGFR (mL/min/ 1.73 m^2)	0.99(0.97,1.01)	0.54
Uric acid (μmol/l)	1.22(0.90,1.66)	0.19
Logarithm of UAE values	1.12(0.88,1.44)	0.36
Metformin (yes)	2.20(1.00,4.86)	0.051
Thiazolidinediones (yes)	0.67(0.30,1.48)	0.32
ACEs and/or ARBs (yes)	0.43(0.20,0.93)	0.032
Statins (yes)	1.56(0.71,3.45)	0.27
Antiplatelet agents (yes)	1.94(0.46,8.16)	0.37

ACE, angiotensin-converting enzyme inhibitor; ARB, angiotensin II receptor blocker; BMI, body mass index; BP, blood pressure; CI, confidence interval; eGFR, estimated glomerular filtration rate; HDL, high-density lipoprotein cholesterol; HR, hazard ratio; UAE, urinary albumin excretion.

Table S11. Summary of hypoglycemic events.

Parameters	Alogliptin treatment group	Conventional treatment group	
Number of confirmed hypoglycemic events			
52 weeks	0(0)	0(0)	-
78 weeks	0(0)	0(0)	-
104 weeks	2(1)	0(0)	0.25
156 weeks	0(0)	0(0)	-
208 weeks	1(1)	1(1)	1.00
260 weeks	1(1)	1(1)	1.00
312 weeks	1(1)	0(0)	1.00
364 weeks	2(2)	2(2)	1.00
416 weeks	1(1)	2(2)	1.00
468 weeks	1(1)	1(1)	1.00
520 weeks	0(0)	0(0)	-
Number of protocol-defined hypoglycemic events			
52 weeks	4(3)	0(0)	0.058
78 weeks	2(1)	6(4)	0.28
104 weeks	0(0)	2(1)	0.50
156 weeks	0(0)	2(1)	0.50
208 weeks	2(2)	2(1)	1.00
260 weeks	1(1)	1(1)	1.00
312 weeks	0(0)	1(1)	0.50
364 weeks	0(0)	0(0)	-
416 weeks	2(2)	3(3)	1.00
468 weeks	2(2)	0(0)	0.50
520 weeks	4(3)	0(0)	0.058
Number of severe hypoglycemic events			-
52 weeks	0(0)	0(0)	-
78 weeks	0(0)	0(0)	-
104 weeks	0(0)	0(0)	-
156 weeks	0(0)	0(0)	-
208 weeks	0(0)	0(0)	-
260 weeks	0(0)	0(0)	-
312 weeks	0(0)	0(0)	-
364 weeks	0(0)	0(0)	-
416 weeks	0(0)	0(0)	-
468 weeks	0(0)	0(0)	-
520 weeks	1(1)	0(0)	1.00

Data are number (%) of patients.
Confirmed hypoglycemia was defined based as plasma glucose ≤3.9 mmol/L. Protocol-defined hypoglycemia was defined based on self-reported probable hypoglycemic symptoms. Severe hypoglycemia was defined as events requiring treatment administration by another person.

Table S12. SPEAD-A trial site investigators (listed in alphabetical order)

Site	Investigator
Jinnouchi Hospital	H Jinnouchi
Jiyugaoka Medical Clinic	H Yokoyama
Juntendo Tokyo Koto Geriatric Medical Center (Department of Medicine, Diabetology & Endocrinology)	S Abe, K Ishida, T Onuma, K Yamashiro, H Yoshii
Juntendo University Graduate School of Medicine (Department of Metabolism & Endocrinology)	H Abe, Y Fujitani, H Goto, T Hirose, F Ikeda, A Kanazawa, M Kawaguchi, K Komiya, T Mita, T Ogihara, C Ohmura, Y Sakurai, F Sato, T Shimizu, M Tamaki
Kansai Rosai Hospital	I Hayashi, T Yamamoto, Y Yamamoto
Misaki Naika Clinic	N Kuribayashi
Naka kinen Clinic	T Osonoi, M Saito, A Tamazawa
Osaka General Medical Center	M Hatazaki, R Kataoka, Y Umayahara
Osaka Police Hospital	K Kosugi, K Sakamoto, K Yoshiuchi
Osaka University Graduate School of Medicine (Department of Metabolic Medicine)	A Imagawa, H Iwahashi, H Kaneto, N Katakami, D Kawamori, T Matsuoka, T Miyatsuka, H Nishizawa, M Ohtsuki
Osaka University Graduate School of Medicine (Department of Metabolic Medicine)	A Imagawa, H Iwahashi, H Kaneto, N Katakami, D Kawamori, T Matsuoka, T Miyatsuka, H Nishizawa, M Ohtsuki
Shiraiwa medical clinic	T Shiraiwa

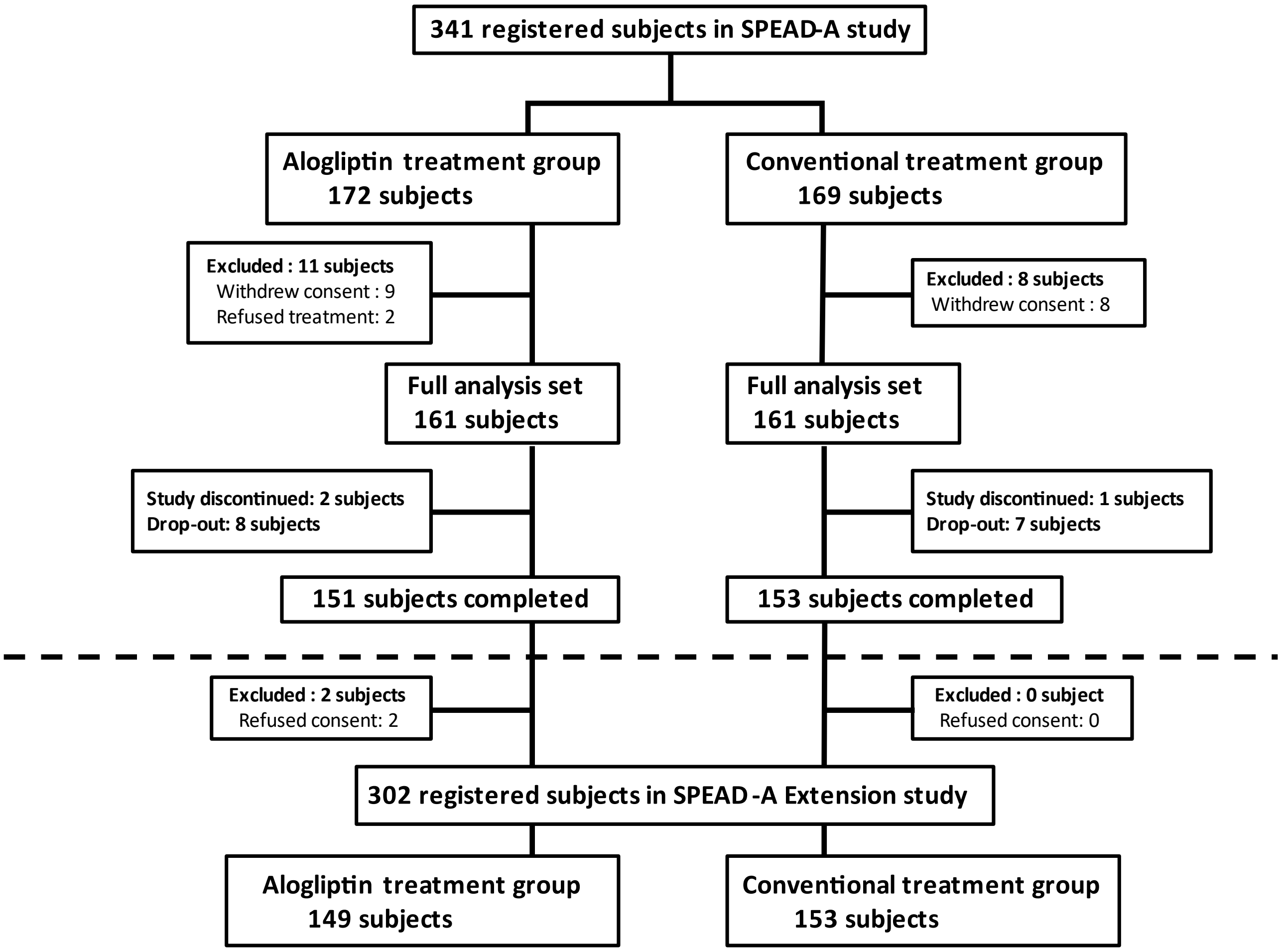

Figure S1. Study flow chart.

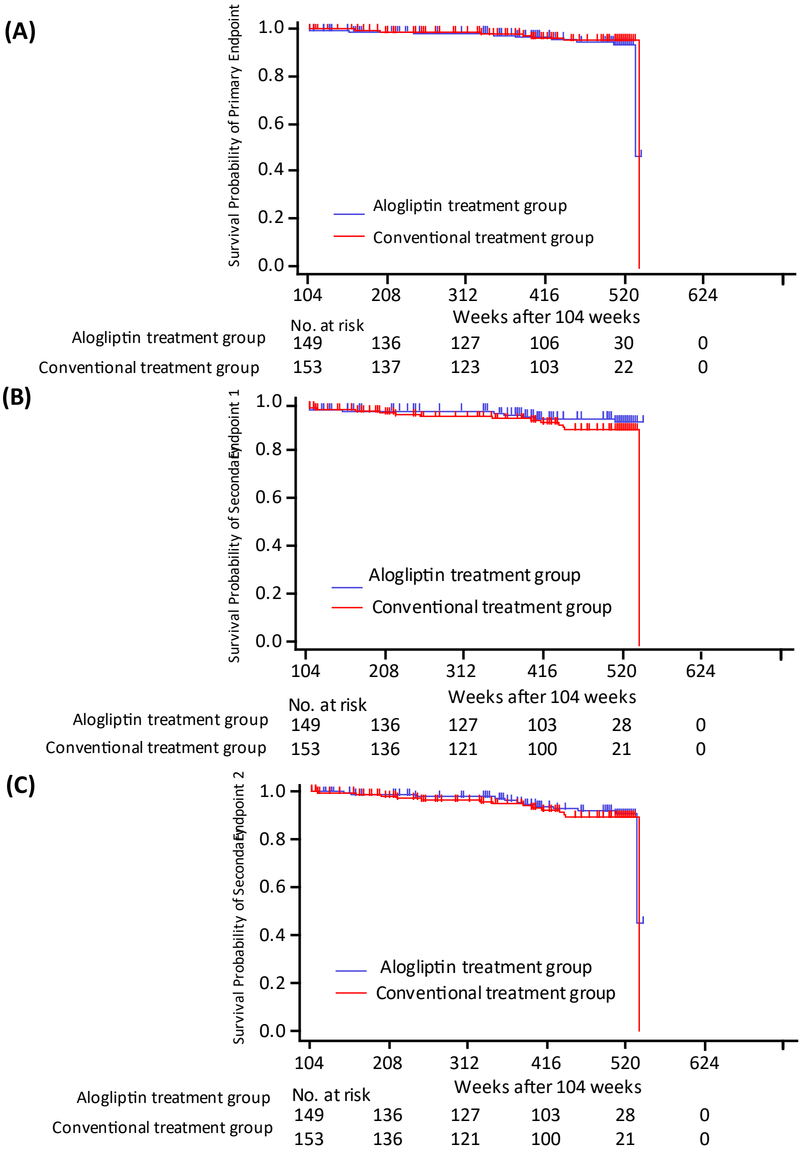

Figure S2. Landmark analysis.

Kaplan-Meier estimates of cardiovascular events.

The figures show Kaplan-Meier survival curves. (A) Primary endpoint. (B) Secondary endpoint 1. (C) Secondary endpoint 2. Blue lines indicate the alogliptin treatment group, and red lines indicate the conventional treatment group.

Primary endpoint: the first occurrence of a major cardiovascular event, which included death from any cause, acute myocardial infarction, and stroke.

Endpoint 1: ischemic heart disease (sudden cardiac death, acute myocardial infarction, hospitalization for unstable angina, and coronary revascularization procedure), cerebrovascular events (ischemic stroke, intracerebral hemorrhage, and subarachnoid hemorrhage), and arteriosclerosis obliterans.

Endpoint 2: Endpoint 1 plus death due to any cause.
